# Do they practise what we teach? A mixed-methods investigation into learning transfer of the maternity team approach in maternity emergencies

**DOI:** 10.1186/s41077-026-00414-1

**Published:** 2026-01-30

**Authors:** Razia Sharif, Yoriko Kikkawa, Sharon Clipperton, Sarah Janssens

**Affiliations:** 1 Mater HealthSouth Mater Misericordiae, Raymond Terrace South Brisbane QLD 4101, Brisbane, Australia; 2 Mater Misericordiae Brisbane Ltd, Mothers Babies and Womens’ Health Services, Brisbane, Australia; 3 University of Queensland Faculty of Medicine, Brisbane, Australia; 4 Adjunct Research Fellow, Griffith Institute for Educational Research, Griffith University, Brisbane, Australia; 5 Senior Research Assistant, Mater Research, Brisbane, Australia; 6 Senior Research Officer, Australian Catholic University, Brisbane, Australia

**Keywords:** Simulation-based training, Maternity emergencies, Teamwork, Learning transfer, Implementation, Maternity team approach, Obstetric education, Non-technical skills, Interdisciplinary training, Qualitative research

## Abstract

**Background:**

Simulation-based training is widely used to improve teamwork in maternity emergencies. However, evidence of learning transfer into clinical practice remains inconsistent. The Maternity Team Approach (MTA)—a structured framework comprising of leadership, role allocation, systematic assessment, and structured recaps—was introduced in a maternity emergency training program at a quaternary tertiary hospital in Australia. This study explored whether the MTA is applied during real-life maternity emergencies and barriers and enablers to its translation in clinical practice.

**Methods:**

A mixed-methods design involving focus groups, interviews, and live clinical observations. Sixty-two maternity clinicians (midwives, obstetricians, and students) participated in eight focus groups and four interviews. Clinical observations of fifty-four live maternity emergencies were conducted by trained raters using an MTA compliance tool. Qualitative data were analysed thematically and triangulated with observational findings.

**Results:**

Participants reported that while the MTA was perceived as useful and beneficial for coordination, communication, and patient experience,however, its application in practice was inconsistent. Structured recaps were most used, while explicit role allocation was less frequently observed. Five key themes influenced MTA uptake: leadership, team culture, team composition, nature of the emergency, and practice norms. Observational data confirmed partial application of MTA elements in clinical settings, particularly in emergencies involving large, unfamiliar, or ad hoc teams.

**Conclusions:**

Despite high perceived value of the MTA, learning transfer into clinical practice is variable and context dependent. Effective leadership, supportive culture, consistent team composition, and alignment between training and real-world conditions are critical enablers. To optimise transfer, maternity emergency training programs should incorporate contextually adaptive strategies and post-training support mechanisms.

**Supplementary Information:**

The online version contains supplementary material available at 10.1186/s41077-026-00414-1.

## Background

Teamwork is essential in maternity emergencies, where time-critical and high-risk situations require coordinated, efficient responses from multidisciplinary teams. Effective teamwork in these scenarios including clear communication, shared decision-making, and coordinated action is associated with improved clinical outcomes [1, 2].

One approach to enhancing teamwork in maternity emergencies is the Maternity Team Approach (MTA). This structured framework focuses on four key principles: allocating leadership, assigning roles, conducting systematic assessments, and performing structured recaps. The MTA aligns with evidence indicating that defined leadership, role clarity, and structured communication improve team performance [1, 2]. While the MTA is currently delivered through local simulation-based education programs, it has not yet been incorporated into national or international maternity curricula. However, similar team-based frameworks have been adopted by other local training initiatives [3].

Simulation-based training is a widely used strategy to build teamwork and clinical skills among multiprofessional healthcare teams. Originating from anaesthesia programs that adapted Crisis Resource Management (CRM) principles from aviation, simulation provides clinicians with opportunities to practise emergency teamwork in a controlled, low-risk environment. Although simulation is strongly recommended for maternity teams, studies evaluating its impact on clinical outcomes have produced mixed results [4–7] highlighting the need to better understand the factors that influence the transfer of learning from simulation to clinical practice [6].

Poor transfer of training is a common issue across healthcare education [8, 9]. Evidence suggests that post-training factors—such as team culture, workplace systems, and individual confidence—may play a greater role in real-world application than the training itself [10]. A variety of individual, team, and organisational factors may influence whether skills learned in simulation are used in practice [5]. Therefore, to understand why training benefits are not always realised in clinical settings, it is necessary to investigate if, and how, simulation-based behaviours are carried over into real-life emergencies. If transfer is limited, identifying the barriers and enablers is essential. Alternatively, if the training approach itself does not sufficiently prepare learners for real-world application, a review of the educational design is warranted.

Our previous work, including course evaluations and staff feedback (unpublished; available on request), indicates that maternity clinicians in our setting value the MTA and report increased confidence in using it after training. However, its application during clinical emergencies has not been formally evaluated. The extent to which the MTA is enacted in practice—and whether training adequately prepares staff to apply the framework in real-world settings—remains unknown. 

### Aim

This study aimed to investigate the application of the MTA by maternity clinicians during real-life emergencies. It also sought to understand clinicians’ perceptions of the MTA’s utility, and to identify the barriers and enablers that influence its use in the clinical environment.

### Methods

This study employed a mixed-methods approach to explore clinicians’ perspectives and gather objective observational data. The research team consisted of three clinicians (SJ, SC, and RS) and one non-clinician educational researcher (YK). RS is a midwife and clinical educator based in the birthing unit, actively involved in both clinical care and education. SJ is an obstetrician who holds clinical and leadership roles within the organisation. SC is a midwifery educator responsible for delivering simulation-based training within a co-located simulation centre. YK, while not clinically trained, is an experienced educational researcher who has observed and studied simulation-based education in healthcare settings.

SJ and SC were involved in the original development of the MTA, and all three clinicians (RS, SC, and SJ) are currently engaged in delivering MTA education. The research team adopted a reflective and reflexive stance throughout the study, recognising how their professional roles and pre-existing involvement with the MTA may have influenced data collection and interpretation. A social constructivist perspective was adopted to explore how the MTA is being applied in clinical practice.

### Study site and the context of the MTA

Mater Mothers Hospital is a quaternary maternity hospital that delivers approximately 10,000 births annually. The hospital operates a mixed public-private model within a shared birthing unit and provides interprofessional education to midwives, student midwives, interns, obstetric registrars and medical students. As is common in the Australian context, midwives provide most of the intrapartum care, collaborating directly with either a specialist for private patients or an obstetric registrar who is overseen and support by a specialist for public patients.

Simulation training for maternity emergencies has been in place since 2013 for midwives and obstetric registrars working within the birth suite and is strongly recommended for student midwives and doctors to attend. Clinicians attend a full-day Maternity Emergency Management (MEM) course onsite in the simulation centre every two years, this course is specific to our institution however, another local institution has adapted this program to suit their context. In 2018, the curriculum was revised to centre on what became known as the *MTA -* a structured teamwork framework adapted from the “pit crew approach” used in resuscitation teams. Grounded in teamwork theory and evidence, the MTA comprises four core elements: (1) assigning leadership, (2) allocating roles, (3) performing a systematic assessment, and (4) conducting a structured recap. These elements were selected as a simplified means of operationalising key Crisis Resource Management (CRM) principles for use in ad hoc maternity teams [11].

The MTA was taught to interprofessional teams through a combination of didactic lectures, group discussions, mental-rehearsal exercises using visually enhanced mental simulation (VEMS), and rapid-cycle deliberate practice in short immersive scenarios. Following this, clinicians were given the opportunities to rehearse the MTA in four immersive simulations, followed by structured, reflective debriefing to consolidate learning.

### Study design and participants

Doctors, midwives, and midwifery students working in the birthing suites at Mater Mothers’ Hospital were invited to participate in focus groups and interviews. Invitation was circulated via staff noticeboards, departmental websites, and email – including to participants of the MEM program. A total of 58 participants (42 midwives, 2 medical students and 14 doctors) contributed to the focus groups and interviews (refer to Appendix 1). Written informed consent was obtained from all participants. This project was reviewed by the local Human Research Ethics Committee and determined to be exempt from full ethical review (QACR/MML/107765).

Staff were also notified via email and noticeboards about the clinical observational component of the study and given the opportunity to “opt out”. A list of those who opted out was provided to all raters, who exited the room when an “opt-out” staff member was identified in the team, with any data collected destroyed.

### Data Collection

To obtain objective data, clinical observations were collected by six trained raters within the midwifery education team, who worked on the birthing unit and attended the room when the emergency alarm was activated. Rater training consisted of three group sessions to evaluate team performance in historically stored simulation videos. During the session, the raters used the MTA compliance tool to assess the team’s application of the four MTA elements on a three-point scale, refined the tool, and reassessed the same videos (see Appendix 2). This process was repeated until consensus was reached. The Clinical Teamwork Scale (CTS), a validated tool of assessing teamwork was part of rater training however, it was ultimately not used for live clinical data collection due to feasibility. The raters struggled to use both the MTA compliance tool and CTS alongside each other in live emergencies. Raters instead prioritised the MTA compliance tool, which became the sole observational measure. A paper-based form was utilised to collect observations of the team, and the data were transcribed into an Excel spreadsheet.

To collect participants’ perspectives, focus groups and interviews were conducted in private rooms on campus by RS. Focus groups were conducted to capture collective perspectives and facilitate discussions among participants with shared experiences. Each session lasted approximately 20 minutes and was held in a private setting within the hospital to ensure confidentiality. A semi-structured interview guide, comprising five open-ended questions, was used to prompt discussion on key topics, including awareness of the MTA, its application in clinical settings, perceived benefits and barriers of the MTA, and suggestions for improving the translation of the MTA into clinical practice.

Individual interviews were conducted with participants who either preferred a one-on-one setting or were unable to attend a focus group session. These interviews provided an opportunity to explore personal experiences and challenges in great depth—particularly those that may not have been openly shared in a group setting. Each interview lasted approximately 30 minutes and followed the same semi-structured format as the focus groups.

All interviews and focus group discussions were audio-recorded with participants’ consent. Audio recordings were transcribed manually by RS with de-identification. Participants were offered access to transcripts to ensure the validity of the transcripts before deleting the audio recordings. Interview and focus group guides were used (see Appendix 3) to provide a structured and consistent approach.

## Data analysis

### Thematic analysis of focus groups and individual interviews

Two researchers who did not conduct observations utilised NVivo software (Lumivero) to conduct thematic analysis of all transcribed texts [12, 13]. Each researcher independently completed their initial analysis phase. They familiarised themselves with the data by reading the text information and using note-taking techniques. They generated initial codes that highlighted the key features of the data by coding the data into initial nodes. To strengthen credibility, both researchers undertook this process independently before meeting to compare interpretations and discuss areas of convergence and divergence. After this initial phase, these researchers and an observer researcher gathered to discuss potential key themes evident within the initial node system. Throughout this stage, the team used reflexive discussion to acknowledge how their professional roles and prior familiarity with the MTA might influence interpretation.

The two researchers again independently conducted the second analysis phase, where they collated their initial nodes into potential themes by gathering all data relevant to each potential theme node. During this phase, YK initially created the thematic map. The team then gathered again to review the map, which was revised accordingly to define and name themes about the participants’ perceptions of experiencing the application of MTA in their clinical practice. An NVivo-based file audit was maintained to support dependability, and participants were offered the opportunity to review their transcripts to ensure accuracy. Finally, the research team determined the final themes and selected vivid examples of texts to report the findings (see Appendix 4-A and 4-B).

## Results

### Overview of findings

Analysis of participant interviews and focus groups aligned with observational data, suggesting a partial implementation of the MTA in clinical practice. Participants reported many perceived benefits of the MTA when it was applied in clinical emergencies. Furthermore, participants articulated the barriers or facilitators to MTA implementation they experienced in their everyday practice. Table 1 presents a summary of themes and subthemes identified from data analysed in the focus groups and interviews. 

**Table 1:** Summary of themes and subthemes

Theme	Subthemes	Description
Leadership	Awareness, confidence, controlling the room, and quality of handover.	Leader familiarity with the MTA strongly predicts whether the framework is initiated and sustained.
Culture	Hierarchy, psychological safety, and department norms.	Hierarchical dynamics influence the willingness to speak up or initiate the MTA.
Team Composition	Familiarity with the MTA, team members, and team size.	Mixed training exposure and rotating teams reduce shared mental models.
Type of Emergency	Pace, familiarity with emergency, and algorithm alignment.	Fast-paced or highly procedural emergencies (e.g. shoulder dystocia) reduce or inhibit MTA use.
Practice Norms	Implicit role allocation, routine responses, disconnect between simulation and reality.	Clinicians default to habitual patterns; recaps adopted more consistently than role allocation

### Clinical teamwork observations

Table 2 presents the mean scores recorded using the MTA compliance tool. Observers’ ratings indicated that leadership allocation and structured recaps were the most consistently applied elements, whereas role allocation was observed less frequently across clinical events. 

**Table 2:** Maternity team approach compliance tool assessment scores

	Post-partum haemorrhage(*n* = 12)	Shoulder dystocia(*n* = 7)	Fetal bradycardia(*n* = 35)	All(*n* = 54)
Leadership established	1.92	1.00	1.74	1.69
Roles Allocated	1.50	1.00	1.29	1.30
Systematic assessment	1.42	1.00	1.46	1.46
Recap using the model	2.00	1.29	1.69	1.70
Total Score	6.84	4.29	6.18	6.15

Each MTA component was score 1–3. A total score (range 4–12) provides an aggregate representation of how completely a team enacted the full MTA. Mean scores highlight which elements were more or less consistently applied. The total score therefore, reflects overall adherence of the MTA, while individual means reveal patterns of behaviour within and across emergency types.

### Interviews and focus groups

The clinical backgrounds and professional roles were varied across focus groups (FGs). FG1, FG5, and FG6 included only midwives; FG7 comprised only doctors; while FG2, FG3, FG4, and FG8 included both midwives and doctors. The composition differences of each group influenced the focus of discussions. For instance, all participants in FG5 were clinical facilitators, and their discussions provided in-depth reflections on the transfer of learning from simulation training to clinical practice (see Appendix 4-A, example D5). In contrast, FG7, which included only doctors, concentrated on the individual responsibilities of team members during emergencies (see Appendix 4-A, example E1). Additionally, student and novice participants emphasised the value of the MTA’s structured approach in supporting their participation and decision-making during high-pressure situations.

“I find it beneficial as a junior to have an external scheme to work through and have something, you know, a structure, like ALS to focus on. (P47, Doctor, FG7.”)

### Current MTA practice and outcomes

Overall, participants reported that the MTA is partially or inconsistently applied during real-life emergencies in the birthing unit, a finding that aligns with the observational data presented in Table 1.

I currently don’t see it used much at all. I see elements of it used in terms of recaps, but even that probably isn’t as often as I’d like. (P57, Doctor, Interview 2)

I feel like we’re almost half and half implementing it. I feel like we’re trying to transition from a task-focused approach to [one that addresses] emergencies rather than the MTA approach. (P51, Midwife, Interview 3)

In particular, the practice of structured recap appeared to be implemented effectively, in contrast to role allocation. The following example of the registered midwife summarises her experience of being involved in maternity emergency training. The practice of recaps became their standard practice after the initial introduction in the simulation training. The example highlighted the success of the everyday implementation of the practice of recaps; however, what could lead to the successful implementation of other components of MTA was unknown.

I [recall] years ago when we did MEM, and we talked about recaps. [Recaps have] always been part of it, and we do the exact thing [when] we come in. We’re motivated. We’re like, recapping everything […] And then, like, it peters off. But now I feel that a recap is standard practice. What has changed in the recap world [is] that we could probably do to the role allocation world. Is it just practice? […] I don’t know what the change is. We recap everything, or we role-allocate everything. (P16, Midwife, FG3)

Despite only partial implementation, the MTA approach was still perceived as beneficial. For example, some participants said that a role-based approach supports practitioner confidence:

I can go on the fundus. I know I can go on the abdomen. I can do this; it makes you more confident walking into an emergency, and then instead of going, I don’t know where I’m meant to be. (P9, Midwife, FG2)

Clinicians also outlined the benefits of the MTA approach to women and families, as well as their own experiences as patients in such emergencies. This aspect highlights that MTA enabled the emergency team to remain calm, resulting in a positive experience for the women [see Appendix 4-B, examples F10 & F11].

Several clinicians also highlighted the MTA’s use of a systematic approach as improving their clinical practice by assisting effective handover (see Appendix 4-B, example F1) in preventing missing any critical aspects via cross-checking (Appendix 4-B, examples F2 and F3), identifying underlying issues (See Appendix 4-B, example F4), helping juniors or novices (see Appendix 4-B Example F5 and F6), and facilitating closed-loop communication (See Appendix 4-B, Example F7).

### Influences on the application of the MTA

Five themes were identified through thematic analysis, focusing on the factors that influence the application of the MTA approach in the clinical environment (see Appendix 4). These themes—(1) leadership, (2) culture, (3) team composition, (4) type of emergency, and (5) practice norms—were interrelated with each other.

The first theme of Leadership was found to be a powerful influence on whether or not the MTA was applied. In the context of MTA, the leader was perceived as the person who initiated role allocation and led the assessment and recap sessions. From the participants’ perspective, the application of the MTA was contingent upon their actions. (see Appendix 4-A example A1 and A2). Most importantly, the participants reported that the leaders’ awareness of the MTA approach and beliefs in its benefits had a huge impact on the MTA application:

It’s very damaging if there’s one leader who doesn’t believe in it and therefore doesn’t do it properly and kind of shrugs her shoulders and says, Oh, I don’t really think this is helpful; that can be really damaging (P57, Doctor, interview 2).

This leadership theme also highlighted several elements that determine leadership quality, which in turn influence the MTA application. It includes (1) situational awareness, (2) confidence and taking ownership, (3) controlling team performance, (4) effective handover, (5) knowledge of the team skillsets, and (6) positioning in the room. This theme was found to be closely aligned within the theme of culture.

It just got to that point where […] everybody felt it was becoming a bit chaotic with lots of people trying me trying to tell the story […]. Dr C was really great and said, “Okay, let’s take a break and recap.” It just brought everybody’s attention, as if we were here now. These are the things we’ve done. Those are the things we need, and we made the woman aware because she knew what we were doing. (P34, midwife, FG 6)

Leaders not only initiate the MTA in specific emergencies but can also influence the culture of practice where team members can speak up freely to initiate MTA elements such as role allocation and recaps. The second theme—***Culture***—outlines the team condition that can either facilitate or prevent the MTA application. The following comment reports the different situations among various departments regarding whether she encourages a leader to adopt the MTA approach or not. A stronger hierarchy or power imbalance was found to be one of the main cultural factors preventing the effective application of MTA.

It’s the culture. It’s not as bad in maternity as in the general adult hospital, where they just follow the doctor’s orders. A lot of them [in maternity] feel like they can stand up. There is that barrier of going, “Oh well, they’re not doing it well. Maybe I’m not going to do it today; therefore, I’m not gonna push him [to use the MTA approach]”. It’s a much bigger picture than just the MTA. It’s much bigger, ingrained in that culture, and it’s like trying to level that hierarchy a little bit so people can speak up. (P31, Midwife, FG5)

The following comments describe another example of different approaches taken by various departments. According to these examples, private patients were treated differently, as their private specialists were more likely to have their own approach, which could not be questioned.

The [private specialists] just take they take ownership of what’s happening (P2, Midwife, FG1)

They might not necessarily follow the same standard procedure that is seen in the public; they have their own thing that they want to do sometimes. They all have their own preferences, they all have their own ways of doing things as well (P9, Midwife, FG2).

In relation to this Culture theme, participants perceived junior staff to be reluctant to exert leadership, and team members also reported feeling unable to speak up or give instructions to other colleagues who may have a closer relationship with the patient (see Appendix 4-A, Examples E4 and E5).

Furthermore, participants felt motivated to deliver MTA if the team environment was welcoming of the MTA application. Conversely, the participants were discouraged if the team did not accept their initiation of the MTA delivery.

Because I have had [my voice being] ignored when I said let’s allocate roles, and then and it just was like, “No”. (P51, Midwife, FG8)

Importantly, each member’s awareness of individual responsibilities was perceived as crucial to creating the culture that improves MTA delivery. The first comment was made when this experienced doctor described his approach to MTA delivery, in which he stepped back from performing clinical tasks to ensure effective leadership. This example also suggests the Culture theme was also strongly aligned with the *team composition* theme. On the other hand, the second comment was made by the experienced midwife, highlighting the MTA application is everyone’s responsibility.

You don’t actually have to be the one doing the vaginal exam or doing the clots because the midwives can do a vaginal exam, or if you’ve got [another doctor] in the room because they have to respond to an emergency buzzer. It’s that learning to step back, and it’s not [all] have to do it yourself? (P46, doctor, FG7)

Verbal prompts do work […] Even if it’s just asking for a recap, that does cut the tension and clarifies what we’re here for, what we’ve done, and what we need to do. I don’t know, bringing in more verbal prompts about the allocation of roles, maybe? Also, I think it’s something that anyone can do right, and you know, doesn’t need to be like the team. We need to ask for a recap that can be provided by anyone in the room. (P39, Midwife, FG6)

While the culture of the unit may have influenced who took on leadership roles and whether they were determined to implement MTA or not in an emergency, Team Composition (the second theme) was also found to have a substantial impact on the application of the MTA. Team composition was described to be highly variable between emergencies. The subthemes of team composition include the team members’ familiarity with MTA and their training experience, the team members’ familiarity with one another, and the number of team members present.

Whether team members had recently attended the MTA training course (MEM) was highlighted as a factor in the use of MTA. The following examples also highlighted the critical nature of training in supporting the culture of applying MTA.

What’s your percentage of people that have been through the [MTA] training? […] If you’ve got a room, your buzzer comes in, and you’ve got duh duh dah, I’m the only person in this room, or two of us have ever been exposed to it as a training resource. Then, how can we expect people to do it? (P34, Midwife, FG6)

If people are like rotating in either like doctors or midwives, they don’t know what this model is. So, I’m not going to implement it because it doesn’t feel safe to do so. (P5, midwife, FG2)

Similarly, the following examples demonstrate that the leaders’ MTA training experience was a primary factor in determining the MTA application, as the leaders shaped the culture in the room. Hence, the strong alignment among MTA familiarity, leadership and culture were underlined.

Some leaders who have recently attended these workshops definitely try to implement those roles. Right, you’re on circulation and the recaps and everything. I’ve noticed that a lot more. (P5, Midwife, FG2)

I noticed that the two team leaders that I did my [training]with. If I have an emergency with them, it works so much differently than with the other team leader because it’s like roles that we rehearsed. (P37, midwife, FG6)

Moreover, Team Size was highlighted as a challenge, as the MTA was difficult to apply when there were too few or too many team members in the emergency, and this was reported to be a dynamic situation (see Appendix 4-A, Example B6-B8 ). The following example described how roles got lost in the morning shift situation where too many staff members came into the room. She emphasised the importance of strong leadership in controlling the appropriate size of the team, which leads to better performance and outcomes for women and their families.

I think that’s where it falls down is that the roles aren’t allocated, particularly on an early shift buzzer goes off. You’ve got 25 people in that room. Most of those [are] not necessary. Unless you’ve got a good leader who kicks people out—I think it’s what’s needed—everyone’s just helping each other out. Which is great, but it’s particularly overwhelming for the family [and] it’s overwhelming for everybody, [be]cause everyone’s [saying, ] “who’s got this, who’s got that”, and roles get lost. […] I’ve seen it with the great leader—[after] everyone’s allocated, people are kicked out, and it works so much better. (P36, Midwife, FG6)

Team members’ familiarity with each other was thought to be another significant factor influencing the MTA application. Working with unfamiliar team members created difficulties in role allocation, as it was unclear who the staff members were or their skill sets. For instance, a novice doctor commented, “If it’s a team [where] I know everyone well, I’ll do it” (P45, doctor, FG7). Conversely, when team members were very familiar with each other, the need for explicit coordination was less, and therefore, the MTA was perceived as being required less (see Appendix 4-A, Example B4-B5).

The theme of *Practice Norms* was found to be a decisive factor in preventing the MTA application. As the experienced midwife (P33, FG5) commented, “*I think there may be a bit of disconnect with sim and MEM and real-life practice.”* The conflict between their everyday routines and the MTA approach was reported by many participants as the disconnect between simulation training at MEM and real-life emergencies, leading to inconsistent application of the approach.

The participants reported that MTA was partially implemented as they adapted the approach to their everyday context. More specifically, while they did not actively verbalise the ownership of leadership and individual roles (i.e., explicit leadership and role allocation), they consciously adapted their individual roles to fit the model and assumed what everyone was doing based on observation (see Appendix 4-A, example D6 and D8). This implicit adoption of roles was described as a common occurrence in real-life emergencies.

I feel like [MTA] did actually work without the verbalisation of people. People went into the roles, and certainly, but nobody said, “I’m on this, I’m on that”. So, they didn’t identify their role, but they certainly did go into those roles (P34, Midwife, FG 6).

The role of implicit application of the MTA was included in the theme of Practice Norms. Many felt that the recap was now a standard expectation in clinical practice (see Appendix 4-A, example D1). Similarly, while role allocation was not commonly explicit, implied allocation of roles was a common and more “natural” way to practice. Explicitly applying the MTA was described as clunky or unnatural (See Appendix 4-A, Example D2-D5) although implicit role allocation was common. Participants also describe the difficulty in changing their standard practice to a new structure, particularly when the perception was that there was no problem with their “old way.”

Sometimes you have to [admit], ‘Oh, this is not really easy.’ This is a different. This is a slightly different algorithm. So, I think practising that and kind of letting go of those other structures I think can be helpful, but it also could be a bit of a challenge (P27, doctor, FG7).

I think it can be clunky […] because some of the PPH which we deal with daily, we kind of just do it. We could do it with our eyes closed; it’s just that we get it, and it goes. So sometimes it feels like it’s almost interrupting the flow to stop and be like, okay, you do [this role], all of those things are being done because it is second nature to us (P53, Midwife, FG4).

The second example above was also coded to the next theme— Type of Emergency. Similar to this example, participants described that common emergencies they knew well, encountered frequently, and performed well in were less likely to be perceived as benefiting from the MTA; therefore, they were less likely to be applied. Conversely, some participants stated that less familiar scenarios were more likely to require an explicit approach, such as the MTA.

I feel like in birth suite, a lot of the time, we automatically just do stuff, you know. Well, I think the classic example of that would be PPH because we see them every single day. So I think we just automatically or fetal bradycardia, we’ve automatically got someone doing blood pressure […] things just start to happen. So I feel like that’s why maybe there’s an assumption, “Yeah. Yeah, we’re doing it”. Whereas maybe it’s something like an epileptic seizure. You know, then we might have to be a bit more specific. (P16, Midwife, FG3)

Certain types of emergencies, where roles aligned well with the algorithm and the pace was slower (such as PPH), were considered ideal candidates for MTA. However, some felt that rapid emergencies and those with a need for role variation, such as shoulder dystocia, were less likely to have the MTA applied (See Appendix 4-A, Examples C1-C4).

In summary, the data from the interviews and focus groups revealed the above complex and interrelated influences on the implementation of teamwork skills learned in the simulated environment. Participants reported partial and adapted implementation of the MTA, which is supported by the team observations.

## Discussion

**Fig. 1:**
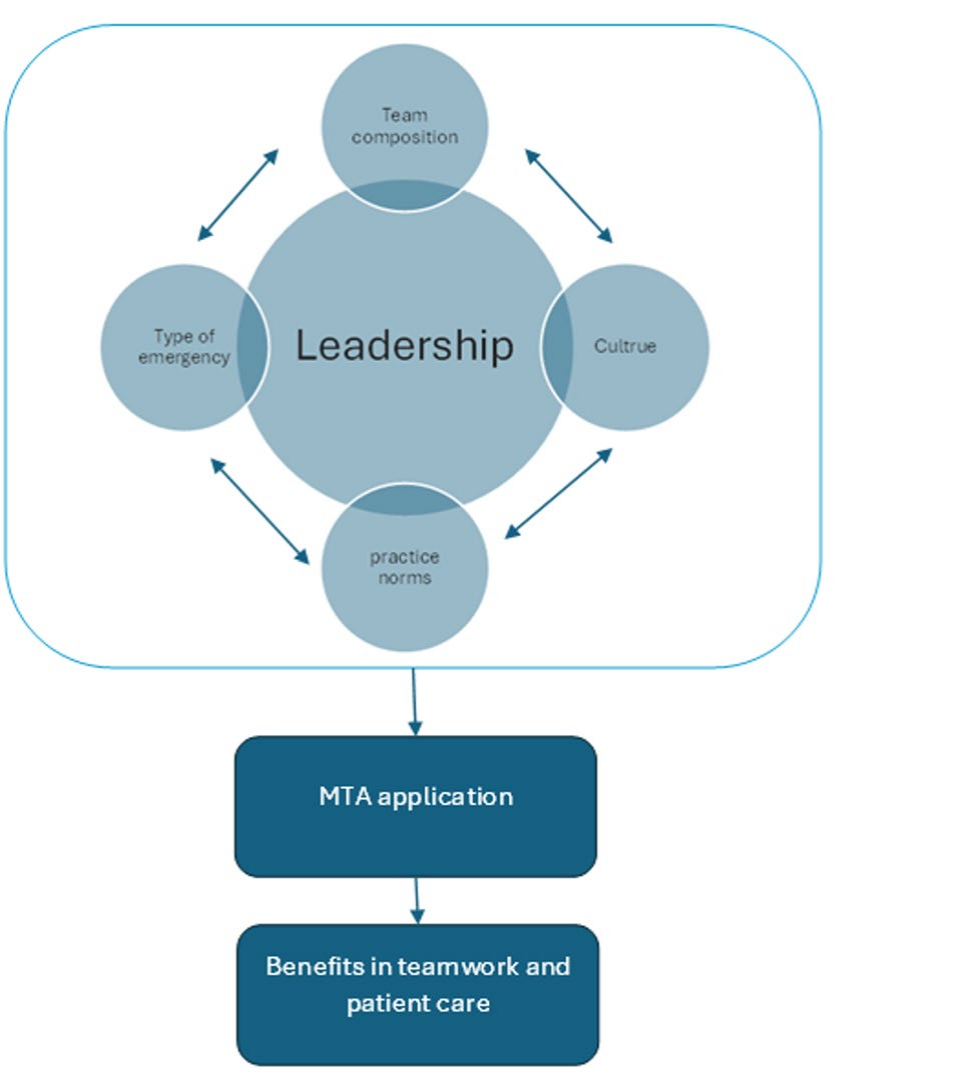
Key themes derived from focus groups and analysis summarises the alignments of key themes emerging from the study. The dominance of leadership as an influencing factor is unsurprising, given the known importance of leadership during medical emergencies [10]. Furthermore, leaders strongly influence training transfer, with supervisor support having been identified in the transfer of training as a significant mediator in the transfer of training into work environments [11]

The study demonstrated that while the MTA is widely recognised by clinicians as a valuable framework for teamwork during real emergencies, its application in practice is often partial, inconsistent and highly context-dependent. The extent to which the MTA is enacted appears to be shaped by a complex interplay of organisational, interpersonal, and contextual factors, which aligns with the broader literature on training transfer, change management and innovation diffusion literature. Fig 1.

Both peer and supervisor support emerged as an essential cultural enabler of implementation. Participants described instances where unsupportive peers or non-adopting leaders discouraged the use of the MTA, ultimately undermining attempts to apply new knowledge and behaviours. The dynamic model of training transfer suggests that such negative feedback loops can lead to individuals to discarding newly learned behaviours, resulting in failed adoption [14]. Conversely, when respected opinion leaders championed the MTA and demonstrated its successful application, other team members were more inclined to adopt the approach. This aligns with change management theory, which emphasises the importance of early adopters and champions in sustaining innovation [15]. Moreover, it highlights that training and post-training interventions should be targeted towards senior clinicians for successful adaptation of the MTA.

Despite the partial implementation of the MTA, there was widespread support for the benefits of the approach. Belief regarding the value and utility of training and the relative advantage of implementing new processes have also been demonstrated to influence the likelihood of training transfer [16, 17]. While there was general support for the utility of the MTA, there was differential belief in the utility of elements such as explicit role allocation and the performance of recaps. Recaps were consistently described as strongly beneficial, explicit role allocation was perceived by some to be often unnecessary, unnatural, and harmful to team performance. In a review of the diffusion innovation, “re-invention” was described as a positive mediator of adoption. When individuals can “adapt, refine or otherwise modify the intervention”, adoption is more likely [17]. The partial implementation of the MTA described in this study may not necessarily represent a failure of transfer but rather an adaptation of the intervention, tailored to the needs of the team – consistent with situated learning in workplace settings [18]. Further investigation is needed to understand if such adaptations, such as implicit role allocation, are helpful or harmful to teamwork in real emergencies, which should then be fed back into the model and training program.

Multiple contextual factors to the specific nature of the emergency and the team represented both positive and negative influences on the use of the MTA by the team. Similarly, how well the MTA aligned with previous training or ways of working specific to team members norms influenced their ability to apply the model [17]. The “innovation-system fit” describes how the degree to which existing norms, strategies and ways of working are aligned with new ways of working influences the assimilation of these new behaviours into routine practice [17]. In other words, the perceived quality compatibility of the intervention of the MTA with various maternity emergency situations. Training should be focused on teaching staff how to operationalise the required adaptations of the model, to ensure compatibility with real-world emergencies. For example, training could explicitly practice with both small and large teams, provide explicit instructions on how to enact the MTA in common emergencies with which the MTA does not easily align, such as shoulder dystocia. Promoting rigid adherence to the model in training may negatively impact staff members’ ability to contextually adapt the model, and flexibility in training should be encouraged [18]. 

Team composition emerged as a critical factor influencing the application of the MTA. The participants consistently reported that the frequent rotations of staff and inconsistent rostering disrupted team continuity and hindered the development of shared mental models (SMMs). SMMs are cognitive frameworks that allow team members to anticipate each other’s actions and work towards a shared goal, facilitating coordinated team performance.[19–21] In the absence of stable team configurations, these cognitive structures are less likely to form, resulting in fragmented communication, reduced situational awareness, and inconsistent application of the MTA framework.

Teams involving rotating staff or individuals without recent MTA training were particularly associated with a reduced likelihood of enacting the framework, as highlighted within the sub theme of team composition. The participants highlighted a lack of shared understanding and inconsistent exposure to MTA principles among team members were significant barriers to its implementation. These findings align with current literature, which suggests that while nontechnical skills, such as communication, leadership and coordination, can be effectively developed through simulation, ongoing and repeated team-based learning is essential to support skills retention and transfer to clinical practice [20].

The presence of new or rotating team members who were unfamiliar with core midwives and doctors on the unit was also perceived to negatively impact psychological safety within the team [22]. Psychological safety – the shared belief that the team is safe for interpersonal risk-taking – is a foundational element of effective team-based care, particularly in high-acuity environments such as obstetric emergencies [23]. In maternity emergencies, time-critical decisions must be made collaboratively, psychological safety enables open communication and adaptive team functioning. However, participants described a reluctance to speak up when unfamiliar or less experienced colleagues were present, particularly if they have not received MTA training. This hesitancy was often rooted in concerns about input would be received, especially in high stress-stress environments where hierarchical norms persist.

These findings align with broader healthcare literature, which consistently links psychological safety to improved clinical decision-making, teamwork, and performance under pressure [22, 23]. In trauma care settings, limited access to team training negatively affected psychological safety and teamwork behaviours, reinforcing the importance of inclusive training models that promote trust, shared expectations, and mutual understanding [21].

In this study, inconsistent access to MTA training emerged as a significant barrier, particularly for new, part-time, or casual staff. These individuals often reported feeling unprepared and unfamiliar with the MTA framework, which contributed to reduced confidence and disengagement during emergencies. Previous research highlights the importance of repeated and inclusive training in developing and sustaining shared mental models across the workforce [20].

Given the well-documented risk of skill decay, especially when clinical opportunities to practise are limited, post-training interventions are essential to embed and sustain behavioural change [10]. Pre- and post-training strategies—such as structured debriefing, reflective practice, and low-fidelity refreshers—have been shown to exert a greater influence on training transfer than the training intervention alone [10]. However, the optimal frequency, content, and delivery format of such strategies remain unclear and warrant further investigation, particularly in the context of resource-intensive, simulation-based education.

This study has several strengths, including a broad and diverse sample of clinicians participating in focus groups and interviews, as well as the triangulation of qualitative findings with observational data. There were limitations related to the collection of observational data, including the inability to collect data using the CTS during real emergencies due to challenges for raters to use both the MTA compliance tool and the CTS simultaneously. Consequently, raters prioritised the MTA compliance tool. Additionally, some observations were incomplete due to late arrival at emergencies or rapid resolution of events, and the emergencies observed may not reflect the usual frequency or variety of emergencies encountered in the birth suite. The absence of race and ethnicity data limits our ability to explore whether MTA implementation influences care equity or patient experiences across diverse groups. Therefore, the observational data should be interpreted with caution.

## Conclusion

This study has demonstrated that while the MTA is perceived to have clear benefits for team coordination and patient care in real-life contexts, its application in clinical obstetric emergencies is shaped by a range of intersecting factors. Leadership, team composition, the nature of the emergency, established practice norms, and underlying cultural and hierarchical dynamics all influence whether and how the MTA is enacted. These findings suggest the need for multifaceted strategies—spanning training, organisational policy, and cultural change—to optimise the integration of structured teamwork models, such as the MTA, into real-world clinical environments.

Future research should focus on implementation interventions that can address these barriers and promote consistent, context-sensitive application of the MTA across maternity care settings. Finally, and importantly, the study has provided key insights into how the application of the MTA may need to be reimagined, particularly in promoting the natural adaptability of high-functioning teams.

## Supplementary Information

Supplementary Material 1.

## Data Availability

The datasets used and analysed during this study are available from the corresponding author upon reasonable request.

## Abbreviation

CRM: Crisis Resource Management
MTA: Maternity Team Approach
CTS: Clinical Teamwork Scale
