# Supplementary material for: Do they practise what we teach? A mixed-methods investigation into learning transfer of the maternity team approach in maternity emergencies
Source: Adv Simul (Lond). 2026 Jan 30;11:15. doi: 10.1186/s41077-026-00414-1 (PMC12930706; doi:10.1186/s41077-026-00414-1)
Supplement: Supplementary file 1 — Supplementary Material 1. [file 41077_2026_414_MOESM1_ESM.docx]

# Appendix 1: Participant Demographics

**Appendix 1-A. Experience of participant altogether**

| Years of Experience | Number of matching cases |
| --- | --- |
| Student | 2 |
| <12 months | 4 |
| 1-5 years | 17 |
| 6-10 years | 17 |
| >10 years | 18 |

**Appendix 1-B Experience of participants_Doctor and Midwife**

|  | Student | < 12 months | 1-5 years | 6-10 years | > 10 years | Total |
| --- | --- | --- | --- | --- | --- | --- |
| Doctor | 1 | 0 | 6 | 5 | 3 | 15 |
| Midwife | 1 | 4 | 11 | 12 | 15 | 43 |

**Table 1-C Individual Codes**

| **Participant ID** | **Focus Group /**  **Interview No** | **Occupation** | **Years of Experience** |
| --- | --- | --- | --- |
| P1 | FG01 | Midwife | > 10 years |
| P2 | FG01 | Midwife | 6-10 years |
| P3 | FG01 | Midwife | 6-10 years |
| P4 | FG01 | Midwife | 6-10 years |
| P5 | FG02 | Midwife | 1-5 years |
| P6 | FG02 | Doctor | 1-5 years |
| P7 | FG02 | Midwife | 1-5 years |
| P8 | FG02 | Doctor | 6-10 years |
| P9 | FG02 | Midwife | > 10 years |
| P10 | FG02 | Midwife | 1-5 years |
| P11 | FG02 | Midwife | < 12 months |
| P12 | FG02 | Midwife | < 12 months |
| P13 | FG02 | Doctor | Student |
| P14 | FG03 | Midwife | 6-10 years |
| P15 | FG03 | Doctor | 6-10 years |
| P16 | FG03 | Midwife | 6-10 years |
| P17 | FG03 | Midwife | < 12 months |
| P18 | FG03 | Midwife | 6-10 years |
| P19 | FG03 | Midwife | > 10 years |
| P20 | FG03 | Midwife | Student |
| P21 | FG03 | Midwife | > 10 years |
| P22 | FG04 | Midwife | > 10 years |
| P23 | FG04 | Midwife | 1-5 years |
| P24 | FG04 | Midwife | 6-10 years |
| P25 | FG04 | Midwife | 6-10 years |
| P26 | FG04 | Midwife | 1-5 years |
| P27 | FG04 | Doctor | 6-10 years |
| P28 | FG04 | Midwife | > 10 years |
| P29 | FG04 | Doctor | > 10 years |
| P30 | FG04 | Midwife | 6-10 years |
| P31 | FG05 | Midwife (clinical facilitator) | > 10 years |
| P32 | FG05 | Midwife (clinical facilitator) | 1-5 years |
| P33 | FG05 | Midwife (clinical facilitator) | > 10 years |
| P34 | FG06 | Midwife | > 10 years |
| P35 | FG06 | Midwife | 1-5 years |
| P36 | FG06 | Midwife | > 10 years |
| P37 | FG06 | Midwife | 1-5 years |
| P38 | FG06 | Midwife | > 10 years |
| P39 | FG06 | Midwife | > 10 years |
| P40 | FG06 | Midwife | 6-10 years |
| P41 | FG06 | Midwife | > 10 years |
| P42 | FG06 | Midwife | > 10 years |
| P43 | FG06 | Midwife | 1-5 years |
| P44 | FG07 | Doctor | 1-5 years |
| P45 | FG07 | Doctor | 1-5 years |
| P46 | FG07 | Doctor | > 10 years |
| P47 | FG07 | Doctor | 1-5 years |
| P48 | FG07 | Doctor | 6-10 years |
| P49 | FG07 | Doctor | 6-10 years |
| P50 | FG08 | Midwife | 6-10 years |
| P51 | FG08 / Int 03 | Midwife (clinical facilitator) | 6-10 years |
| P52 | FG08 | Midwife | 1-5 years |
| P53 | FG08 | Midwife | 1-5 years |
| P54 | FG08 | Midwife | < 12 months |
| P55 | FG08 | Doctor | 1-5 years |
| P56 | Int01 | Doctor | 1-5 years |
| P57 | Int02 | Doctor | > 10 years |
| P58 | Int04 | Midwife | > 10 years |

# Appendix 2: Compliance with Maternity Team Approach

# Date: ____________________________________

# Emergency attended:  Fetal bradycardia / Shoulder dystocia / Postpartum haemorrhage /

# Notes:_____________________________________________________________________________________________________________________________________________________________

# Circle observed actions / behaviours in line with Maternity Team Approach.

| Assessment | 1 | 2 | 3 |
| --- | --- | --- | --- |
| Was leadership allocated at commencement of team arrival? | No clear leader established | Implied leadership and leads inconsistently | Verbal declaration of leadership and leads consistently |
| Were roles allocated? | No or minimal role allocation evident | Some roles implied and assumed but not adhered to for no apparent reason | Roles allocated and mostly adhered to unless good reason to change |
| Did a systematic assessment occur using the model? | No systematic assessment | Partial systematic assessment, or assessment not aligned with model | Systematic assessment using the model |
| Did a recap occur using the model? | No recap | Recap but not utilising the model, or incompletely using the model | Recap occurred using the model |

# Total Score: ___________________________________

# Rater name: ___________________________________

# Appendix 3: Interview Guide for Focus Groups and Interviews

Check consent confirmed [ ]

Recording on [ ]

Participant details confirmed [ ]

1. Are you familiar with the Maternity team (stick chick) approach in maternity emergencies?

- If NO
  - what suggestions do you have to improve teamwork in maternity emergencies at Mater?
- If YES
  - what are your thoughts on this approach to emergencies?
  - Tell me more about that

1. To what extent do you see the Maternity team approach implemented when emergencies arise in childbirth at Mater?
   - Are there times when it is more or less likely to be used?
   - Why do you think this is?
2. What do you see as the benefits and downsides of the maternity team approach?
   - Can you tell me more?
   - Are there other pros or cons?
3. What helps staff to use the maternity team approach?
   - Is there something we can do to augment this further?
4. What prevents staff from using the maternity team approach?
   - Is there something that can be done to over come these barriers?

Thanks staff for participation. Reminder of ability to withdraw consent.

## Appendix 4: Themes

**Appendix 4-A. Influence Themes, Subthemes, Quote Examples, and Thematic Alignments**

| Influence Theme | Subthemes | Example ID | +/- | Examples | Alignment to other themes |
| --- | --- | --- | --- | --- | --- |
| Leadership | N/A | A1 | + | I think if there's a strong approach from [the team leader], everyone else will follow suit with the approach. (speaker 2, midwife, FG4) |  |
|  |  | A2 | - | If no one steps into that role [as leader], then the MTA doesn't happen (P46, doctor, FG7) |  |
|  |  | A3 | - | The approach of some of the team leaders, maybe, there may be some conflict. And maybe junior staff are not able to stand up to the team leader, depending on who the team leader is. So it's not just the doctors, it's maybe the some of the team leaders that they maybe a barrier. (P33, Midwife, FG5) | Culture |
|  |  | A4 | + | I noticed that the two team leaders that I did my MEM with. If I have an emergency with them, it works so much different than it at the other team leader because it's like roles that we rehearsed. (P37, midwife, FG6) | Team Composition (MTA familiarity), Culture |
|  |  | A5 | - | Some leaders who have come to these workshops recently, definitely try to implement those roles. Right you're on circulation and the recaps and everything. I've noticed that a lot more. (P5, Midwife, FG2) | Team Composition (MTA familiarity); Culture |
| Team composition | MTA familiarity | B1 | - | What's your percentage of people that have been through the [MTA] training? […] If you've got a room, your buzzer comes in and you've got duh duh dah, I'm the only person in this room or two of us have ever been exposed to it as a training resource. Then, how can we expect people to do it? (P34, Midwife, FG6) | Culture |
|  |  | B2 | - | If people are like rotating in either like doctors or midwives, they don't know what this model is. So I'm not going to implement because it doesn't feel safe to. (P5, midwife, FG2) | Culture |
|  | Member familiarity | B3 | + | If it's a team [where] I know everyone well, I'll do it. (P45, doctor, FG7) | Culture |
|  |  | B4 | - | When you're in the birth suite and you work with those staff, really, frequently, you're very aware of their capabilities [and] their skill set […] The leader can almost take a bit more of a step back because a lot of it is implied. (P28, midwife, FG4) | Leadership |
|  |  | B5 | - | I actually think the more familiar you are with your team the less we actually do [the MTA] (P24, Midwife, FG4) | Culture |
|  | Team size | B6 | - | I think it's something else that can be difficult is when you press the emergency bell. You're not entirely sure how many team members you’re going to get. (speaker 8, midwife, FG8) |  |
|  |  | B7 | +/- | So I think sometimes we need to get better at saying, like, if you don't have a role, then you don't need to be here. (interview 4, midwife) |  |
|  |  | B8 | - | We don't always have enough people like especially overnights and stuff. So like you, potentially you you're already doing like two or three roles (P7, Midwife, FG2) |  |
| Type of emergency | N/A | C1 | - | It’s a really good model but doesn’t always works in situations where the emergency might be swift. (P32, Midwife, FG5) | Practice norms |
|  |  | C2 | - | I definitely find shoulders dystocia do not have any role allocation at all (P51, Midwife, Interview 3) | Practice norms |
|  |  | C3 | +/- | Yeah, I think I, I yeah, I see it less used in a shoulder dystocia. Yeah. And I guess that's probably as the TL responding you, you need to get hands on. (P2, Midwife, FG1) | Practice norms |
|  |  | C4 | + | Say, a maternal collapse, yeah, or something different. I feel like people would be a bit more like “What’s my role?”. (P35, Midwife, FG6) | Practice norms |
| Practice norms | Becoming standard practice | D1 | + | So I remember years ago when we did MEM, when we talked about recaps, always been part of it, and we do the exact thing we come in and. We're motivated. We're like, recapping everything. You know, like everything. And then you're right. Like it peters off. But now I feel like recap is standard practise. | Culture, team composition |
|  | Not natural way/ disconnection | D2 | - | I feel like [MTA] did actually work without the verbalisation of people. People went into the roles and certainly, but nobody sort of said “I'm on this, I'm on that”. So, they didn't identify their role, but they certainly did go into those roles (P 34, midwife, FG 6) | Culture |
|  |  | D3 | - | I think there may be a bit of disconnect with sim and MEM and real-life practice. (P33, CF, FG5) | Culture |
|  |  | D4 | - | We were often taught, as the doctors, ABC approach. in a very harsh way. We start doing obstetrics, you get taught the 4T’s and 4 Hs and the new stuff—the RANZCOG way overstate monitor or whatever, so that sometimes it feels like we're so bombarded with structures on how [to] approach [to an] emergency. [But] they're all the same. They all cover the same content in the end, but they're slightly different acronyms and slightly different ways of following it. I do wonder if that stick chick works really effectively and it's metrics. I think it goes down well and because like drugs is something that is not named after a CD, it's a really effective part of resuscitation and management. But it does mentally. Sometimes you have to be like, oh, this is not really easy. This is a different. This is a slightly different algorithm. So I think practicing that and kind of letting go of those other structures I think can be helpful, but also could be a bit of a challenge. | Culture |
|  |  | D5 | - | Because I guess at the minute people would earn to the MEM might once or once a year, or once every two years, so they get it from there. But as soon as they've got the knowledge, if they come back and they have they don’t see anyone else using that same knowledge then they’re just going to not use it. (P31, Midwife, FG5) | Culture; Team Composition (MTA familiarity) |
|  | Assumptions and routines | D6 | - | I don't think I've seen people clearly call it like calling or being allocated roles, but people do gravitate towards the roles (P50 Midwife, FG8) | Culture |
|  |  | D7 | - | There's a lot of self-allocation where people kind of also gravitate to where they feel most comfortable. So I think that's how sometimes the roles are more allocated (P4, Midwife, FG1) | Culture |
|  |  | D8 | - | So it’s a lot of not very clear role allocation, assumed team leaders and then your recaps. So it’s kind of like let’s not do this, but we'll do this part of the MTA (P51 CF, FG 8) | Culture |
|  |  | D9 |  | The approach of some of the team leaders, maybe, there may be some conflict. | Culture. Leadership |
| Culture (and hierarchy) | Individual responsibilities | E1 | + | You don't actually have to be the one doing the vaginal exam or doing the clots because the midwives can do a vaginal exam, or if you've got your 6611 in the room because they have to respond to an emergency buzzer and it's that learning to step back and it's not have to do it yourself? (P46, doctor, FG7) | Leadership, team composition |
|  |  | E2 |  | Verbal prompts do work […] Even if it's just asking for a recap that does just cut the tension and OK what are we here what have we done what do we need to do? I don't know bringing in more verbal prompts about allocation of roles maybe? Also I think is something that anyone can do right and you know doesn't need to be like the team we need to asking for a recap that can be anyone in the room. (P39, Midwife, FG6) |  |
|  | Power imbalance | E3 | - | I felt like I could have taken charge, but I felt [...] I was too junior (P32, Midwife, FG5) | L |
|  |  | E4 | - | When you come to answer a buzzer, you don't want to feel like you're stepping on the toes of the primary midwife (P53, Midwife, FG8) |  |
|  |  | E5 | - | I think you feel a bit awkward.... It's probably not within our nature to go–“Mary do this” (P2, Midwife, FG1) | Leadership |
|  | Different department | E6 |  | It's the culture. It's not as bad in maternity as in the general in the adult hospital where they just follow the doctor's orders. A lot of them [in maternity] feel like they can stand up. There is that barrier of going “Oh well, they're not doing it well. Maybe I'm not going to do it today; therefore, I'm not gonna push him [to use the MTA approach]”. It's a much bigger picture than just the MTA. It's much bigger, ingrained in that culture, and it's like trying to level that hierarchy a little bit so people can speak up. (P31, Midwife, FG5) | Leadership |
|  |  | E7 | - | The [private specialists] just take they take ownership of what’s happening (P2, Midwife, FG1) | Team composition, Leadership |
|  |  | E8 | - | They might not necessarily follow the same standard procedure that is seen on like the public...they have their own thing that they want to do sometimes do. They all have their own preferences, they all have their own ways of doing things as well (P9, Midwife, FG2) | Team composition, leadership |

**Appendix 4-B. Current MTA Themes, Quote Examples, and Thematic Alignments**

| Current MTA Theme | Example ID | +/- | Examples | Alignment to other themes |
| --- | --- | --- | --- | --- |
| Effective handover | F1 | + | I had a PPH yesterday with a senior registrar, and she clearly did a recap, went through everything and it was really helpful, as things were settling down in that PPH to have, “This is what drugs have gone through. This is what we've done. This is our EBL...” and [she] really went through it quite concisely. And I think that works really well. (P50, midwife, FG 8) | Emergency type, leadership |
| Cross-checking | F2 | + | [It is] systematic so nothing gets missed and there's no duplication, because if there's duplication or confusion then there's lots of crosstalk, particularly in sort of replicated evolve in emergencies and can get noisy. So if everybody has a role, we know what we can see it in our mind, it just helps to clarify the situation in real life. (P45, Doctor, FG7) | Maintain calmness |
|  | F3 | + | I think the benefit is that it gives you an opportunity to check if things have been missed. So you have that like flow chart in your head, I suppose that you go have we given all the appropriate medication? Have we done this? (P2, Midwife, FG 1) |  |
| Identify underlining issues | F4 |  | Rather than just focusing on the emergency, you know the diagnosis saying, “Ok, what else is going on and the recap”. I think the recap is crucial […] It gives you a chance to focus on every aspect of emergency and not just the emergent issue. It helps you to identify any underlying issues. (P2, Midwife, FG1) |  |
| Assist juniors | F5 | + | Coming from like a staff side of this as graduate who is quite junior. Having the roles assigned really helps when you haven't got a lot of experience in emergency situations because sometimes you actually just need someone to say, hey, are you happy to jump on pelvis? And it's like actually, yes, I am capable of doing that. But if no one says anything and everyone kind of just comes in and assumes a role and there's no communication about who's on that role you end up kind of just being pushed back to the side and then when everyone disperses and leaves afterwards. You're kind of like, cool, like I was not really a part of it. I don't quite know what happens, but now I have to document. (M26, Midwife, FG4) |  |
|  | F6 | + | I find it beneficial as a junior to have an external scheme to work through and have something, you know, a structure, like ALS to focus on. (P47, Doctor, FG7) |  |
| Closed-loop communication | F7 | + | I think it really helps to close that loop of communication. One person is on that [particular role], and then you say, “I'm on drugs, this is what's been given” instead of being like who’s done that. […] Like [this] person has [this] defined role and can just close that loop of communication. (P25, Midwife, FG4) |  |
| Maintain calmness | F8 | + | I think it takes panic out of the room because everyone kind of learned figures very quickly what they're doing and people settle down and not worried about things that they least as much (P46, Doctor, FG7) |  |
|  | F9 | + | It just got to that point where […] everybody felt it was becoming a bit chaotic with lots of people trying me trying to tell the story […] and Dr C was really great and like, “OK, let's take a break and recap”, it just brought everybody's attention like we are here now. These are the things we've done. Those are the things we need and made the woman aware because like she knew what we were doing. (P34, midwife, FG 6) | Leadership/ Calmness |
| Patient Benefits | F10 | + | If I was the woman, it would feel like, so much more calm. I think it would be less dramatic and less kind of scary when everyone has a defined role, you know, and you have that one person up with you. (P25, Midwife, FG 4) | Maintain calmness |
|  | F11 | + | This woman had a [previously] very traumatic experience, so we spent a lot of time antenatally [this time] talking about the policy and debriefing with her experience .. like we literally talked her through about [the MTA…] It felt that she was much prepared this time […] powerful experience for her. She was like, “I was very happy with everything”. (P18, Midwife FG6) |  |
